# Development of an anti-oxidative intraocular irrigating solution based on reactive persulfides

**DOI:** 10.1038/s41598-022-21677-4

**Published:** 2022-11-10

**Authors:** Hiroshi Kunikata, Hiroshi Tawarayama, Satoru Tsuda, Takaaki Akaike, Toru Nakazawa

**Affiliations:** 1 Department of Ophthalmology, Tohoku University Graduate School of Medicine, 1-1 Seiryo-machi, Aoba-ku, Sendai, 980-8574 Japan; 2 Department of Retinal Disease Control, Tohoku University Graduate School of Medicine, Sendai, Japan; 3 Department of Environmental Health Sciences and Molecular Toxicology, Tohoku University Graduate School of Medicine, Sendai, Japan; 4 Department of Advanced Ophthalmic Medicine, Tohoku University Graduate School of Medicine, Sendai, Japan; 5 Department of Ophthalmic Imaging and Information Analytics, Tohoku University Graduate School of Medicine, Sendai, Japan

**Keywords:** Diseases, Health care, Medical research

## Abstract

Anti-oxidative intraocular irrigating solutions (IISs) based on reactive persulfides, such as oxidized glutathione disulfide (GSSG), are commonly used worldwide. However, even with GSSG-based IISs, it has been shown that oxidative stress can occur during surgery, posing a risk to intraocular tissues. This study compared two IISs: one containing GSSG and one containing an oxidized glutathione trisulfide (GSSSG). Experimental in vivo irrigation with the IISs in rabbits showed that there was less leakage into the anterior chamber of rabbit serum albumin during perfusion with a 300-μM GSSSG IIS than with a 300-μM GSSG IIS. Experimental in vivo cataract surgery in rabbits showed that aqueous flare was suppressed 3 days after surgery with a 600-μM GSSSG IIS, but not with a 300-μM GSSSG or 300-μM GSSG IIS. Furthermore, an in vitro experiment, without any live tissue, showed that reactive oxygen species were suppressed more strongly with a 600-μM GSSSG IIS than with a 300-μM GSSG IIS. Thus, this study found that novel IISs based on GSSSG had anti-inflammatory and anti-oxidative effects during and after intraocular surgery and may decrease the rate of complications after surgery.

## Introduction

Intraocular surgery is becoming increasingly common around the world, particularly for the extraction of cataracts, which are still by far the most common cause of blindness^1^. More than 20 million cataract surgeries are performed per year worldwide, most of them using phacoemulsification, a technique that has been shown to generate reactive oxygen species (ROS), leading to the potential risk of oxidative stress-induced damage to intraocular tissues^2–4^. Modern intraocular surgery always requires the intraoperative use of an intraocular irrigating solution (IIS). Oxidized glutathione disulfide (GSSG) is known to exert a protective effect on intraocular tissues^5–7^. GSSG-based IISs were introduced to reduce oxidative stress-related injury to ocular tissues, i.e., to prevent breakdown of the blood-aqueous barrier (BAB), maintain corneal clarity by protecting endothelial cells, and prevent inflammatory macular complications. However, even though GSSG seems to be effective in reducing oxidative stress during phacoemulsification, it has now been in use for 20 years, and there are still reports of decreased corneal endothelial cell density, corneal edema, and bullous keratopathy after procedures using phacoemulsification^8–13^. All of these corneal complications are related to endothelial cell injury, which is at least in part caused by the free radicals produced during the procedure. Since in vivo mitosis of corneal endothelial cells is practically non-existent, lost cells are not replaced, and if their density drops below a critical level, corneal edema will occur^14,15^.

High-intensity ultrasound oscillation is known to produce free radicals in solution^16^, and phacoemulsification has been reported to produce free radicals in animal eyes, leading to corneal endothelial damage^17^. Intraocular surgery thus tends to cause high levels of oxidative stress, and this tendency persists even with the use of current GSSG-based IISs. Oxidative stress has been shown to cause corneal endothelial disorders during cataract surgery^4,9,18^ and lens opacity or cataract progression after lens-sparing vitreous surgery^12,19^. Oxidative stress also increases in the retina after vitreous surgery^20^. Thus, it is necessary to establish new IISs based on endogenous antioxidant systems that are safe, mitigate oxidative stress, and more effectively protect intraocular tissues.

The classical antioxidant system is already well known; it includes catalase, glutathione peroxidase, superoxide dismutase, glutathione, cysteine, and polyphenols. However, recent research on reducing oxidative stress has suggested novel antioxidant systems and new approaches to formulating IISs^21,22^. This research has focused on reactive sulfur molecular species, which comprise what is termed the “new antioxidant system”^21^. Various reactive persulfides and polysulfides have been reported to form endogenously in mammalian cells and tissues, including human plasma^21,22^. These molecules have been reported to be present in the eye, and have been found to have potent antioxidant and redox signaling functions^22^. Furthermore, some reactive sulfide species that have a protective effect against oxidative stress are upregulated in the aqueous and vitreous humors of diabetic eyes^22^. Based on these findings, the present study set out to develop an advanced IIS based on oxidized glutathione trisulfide (GSSSG), a promising and stable polysulfide molecule. We investigated the ability of this IIS to act as a scavenger of ROS and protect intraocular tissues.

## Methods

### Animals

10- to 12-week-old male Slc:JW/CSK rabbits were obtained from CLEA (Tokyo, Japan) and maintained at animal facilities at the Tohoku University Graduate School of Medicine under a 12-h light/dark cycle. Animal anesthesia was performed by subcutaneous injection of a mixture of ketamine and xylazine, following pre-anesthesia with isoflurane.

All experimental protocols were approved by the Committee on Animal Research at Tohoku University. All methods are reported in accordance with the ARRIVE guidelines (https://arriveguidelines.org) and were carried out in accordance with relevant guidelines and regulations.

### In vivo detection of rabbit serum albumin after anterior chamber irrigation in rabbits

Fluorescein isothiocyanate (FITC) labeling of rabbit serum albumin (RSA) was performed with a procedure described previously^39^. Briefly, RSA (Sigma-Aldrich, St. Louis, MO, USA) was reacted with FITC isomer I-celite (Sigma-Aldrich) in 0.2 M carbonate-bicarbonate buffer (pH 9.4). The mixture was then centrifuged to collect the supernatant, which contained both FITC-labeled and unlabeled RSA. Separation of FITC-labeled RSA and exchange of the carbonate-bicarbonate buffer with 0.1 M phosphate buffered saline (at pH 7.4) were performed using a Sephadex G-25 medium (Sigma-Aldrich)-filled filtration column.

Concentration of the FITC-labeled RSA solution was adjusted to 80 mg/ml with a physiological salt solution (Otsuka, Tokyo, Japan). FITC-labeled RSA (250 mg, corresponding to 3.125 ml) was administered via the posterior auricular vein of the rabbits 10 min before the experiments. Two 26G needles were inserted into the anterior chamber. Part I of the BSS PLUS 500 Intraocular irrigating solution 0.0184% (BSS PLUS diluent; Alcon, Fort Worth, TX, USA) containing GSSSG, GSSG, or phosphate buffer (PB) as a control was actively flowed at 1.5 ml per minute for 60 min (for a total of 90 mL) through one of the needles using a peristaltic pump (Tokyo Rikakikai, Tokyo, Japan). The irrigating solution was passively flowed out through the other needle and pooled in sample tubes every 10 min to measure the concentration of FITC-labeled RSA.

A direct sandwich enzyme-linked immunosorbent assay (ELISA) using horseradish peroxidase (HRP)-conjugated detection and capture antibodies for FITC was used to determine the concentration of FITC-labeled RSA in the irrigating solutions. Briefly, immunoplates (Thermo Fisher Scientific, Waltham, USA) were coated with the capture antibody (M228-3; 1:1000 dilution; MBL, Nagoya, Japan) at 4 °C overnight. The collected irrigating solutions were applied to the coated plates and incubated at 4 °C overnight. After extensive washing, the detection antibody (ab6656; 1:10,000 dilutions; Abcam, Cambridge, UK) was applied to the plates. Color development was performed using a TMB microwell peroxidase substrate (Seracare, Milford, MA, USA). Signal intensity was measured at 450 nm using a SpectraMax M2e microplate reader (Molecular Devices, San Jose, CA, USA).

### In vivo detection of aqueous flare after cataract surgery in rabbits

A corneal tunnel incision was made, followed by phacoemulsification and aspiration, in both eyes of each rabbit. During the procedure, a BSS PLUS diluent containing GSSG (300 µM), GSSSG (300 µM), or GSSSG (600 µM) was used in one eye, and a PB-containing BSS PLUS diluent was used as a control in the other eye. Surgical time differed in the eyes that underwent the procedure, but the total volume of irrigating solutions used during surgery was less than 50 ml per eye in all cases. The glutathione-containing solutions were administered during, but not after, cataract surgery. All surgeries were performed by the same doctor (H.K.), with the surgery in the second eye performed immediately after the surgery in the first eye.

Cataract surgery was performed with a standardized technique based on the Alcon Accurus 800CS Phacoemulsifier (Alcon Surgical, Fort Worth, TX, USA). An ophthalmic viscosurgical device (OVD; Opegan High, Santen Pharmaceutical, Osaka, Japan) that contained 1.0% hyaluronic acid was injected via the 2.2-mm corneoscleral limbal incision into the anterior chamber. Capsulorhexis and hydrodissection were performed through the limbal incision. The lens nucleus was removed using the following settings: ultrasound power in conventional mode, 40%; vacuum, 100–200 mmHg; and irrigation bottle height, 60 cm, followed by complete removal of the OVD. We did not implant an intraocular lens in any eye.

Postoperatively, topical antibiotic ointment was instilled into the eyes. Anterior chamber flare was measured with a laser flare meter (Kowa FM-600; Kowa, Aichi, Japan) preoperatively and at postoperative days 1, 3, and 7.

### In vitro detection of phacoemulsification system-induced ROS production

A ROS detection probe, hydroxyphenyl fluorescein (HPF; Goryo Kagaku, Sapporo, Japan), was used to quantify ROS production. The HPF probe was diluted with a BSS PLUS diluent at a final concentration of 5 µM. Furthermore, GSSSG or GSSG (Kyowa Hakko Bio, Tokyo, Japan) was added to the HPF-containing solution at various final concentrations. A PB-containing BSS PLUS diluent was used as a control. Ultrasonication was performed using the handpiece of the Alcon Accurus 800CS Phacoemulsifier at 40% power for 60 s. Fluorescence intensity was measured at 535 nm (excitation: 485 nm) using a SpectraMax M2e microplate reader.

## Results

### GSSSG-mediated protection of the BAB in rabbits

**Figure 1:**
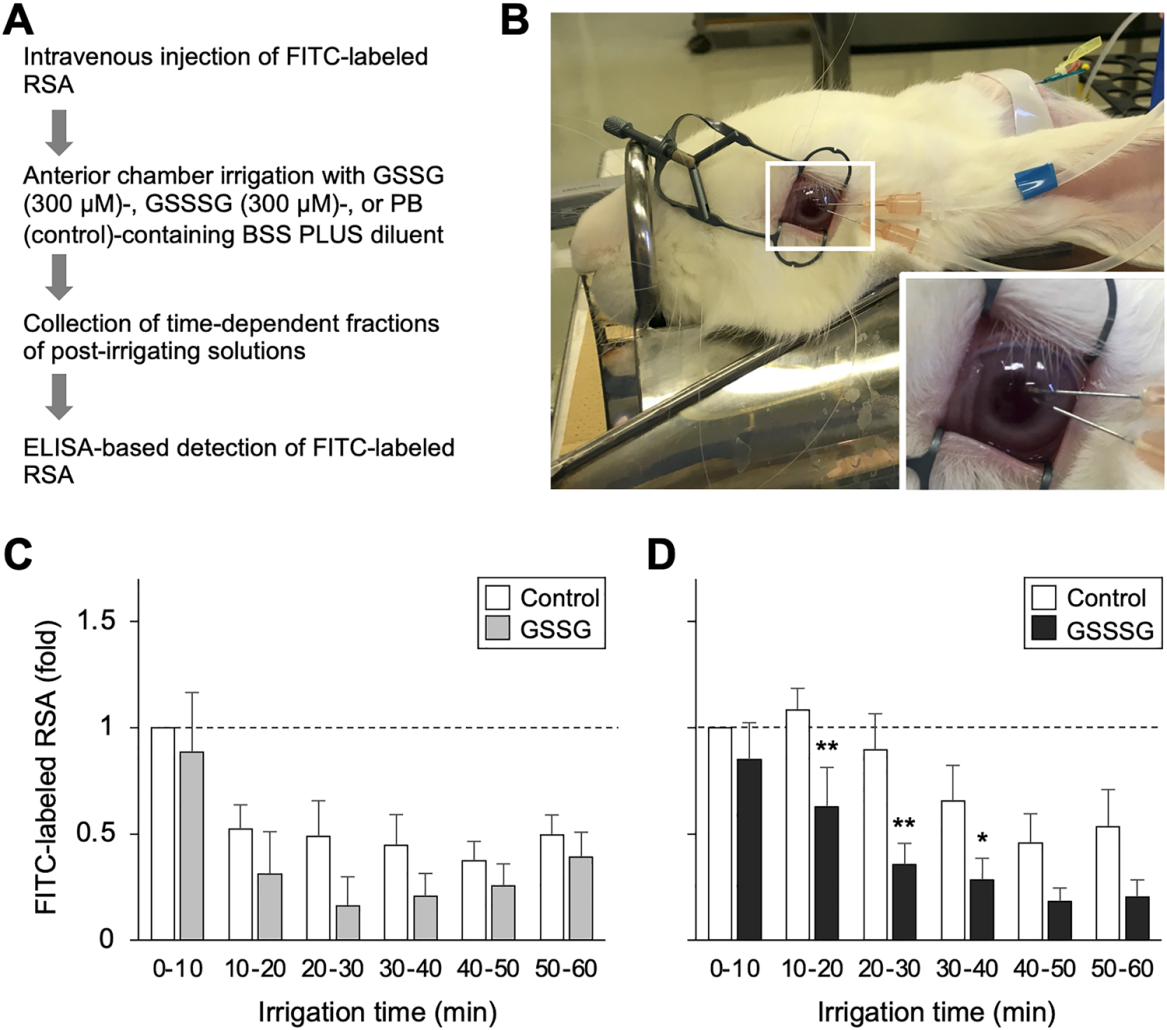
Effect of GSSSG on protection of the BAB. (**A**) Experimental procedure for quantification of BAB permeability. (**B**) Photographs of a rabbit undergoing anterior chamber irrigation. In the box, a close-up of the ocular area is shown. (**C**, **D**) ELISA-based quantification of FITC-labeled RSA in solution containing GSSG (300 µM) (**C**) or GSSSG (300 µM) (**D**) after perfusion in the anterior chamber. The concentration of FITC-labeled RSA in the solutions, collected at the shown irrigation time, is shown as a relative value to that in the control solution collected between 0 to 10 min during perfusion. Error bars indicate standard error of the mean. *P < 0.05 and **P < 0.01 (two-way repeated-measures ANOVA; n = 8).

We examined the effect of GSSSG on the permeability of the BAB using anterior chamber irrigation in rabbits. The rabbits received an intravenous administration of FITC-labeled RSA and then underwent irrigation with a solution containing GSSSG (300 µM), GSSG (300 µM), or PB (as a control) through a sharp needle (Fig. 1A,B). The irrigating solution was then collected in a time-dependent manner, and the FITC-labeled RSA concentration was quantified with ELISA and a FITC antibody (Fig. 1A). The concentration of FITC-labeled RSA in the GSSG-containing solution and the control solution was similar, and neither was at a significant level (Fig. 1C). In contrast, GSSSG-containing solution significantly prevented leakage of FITC-labeled RSA into the irrigating solution (Fig. 1D).

### Inhibitory effect of GSSSG on increased aqueous flare after cataract surgery in rabbits

**Figure 2:**
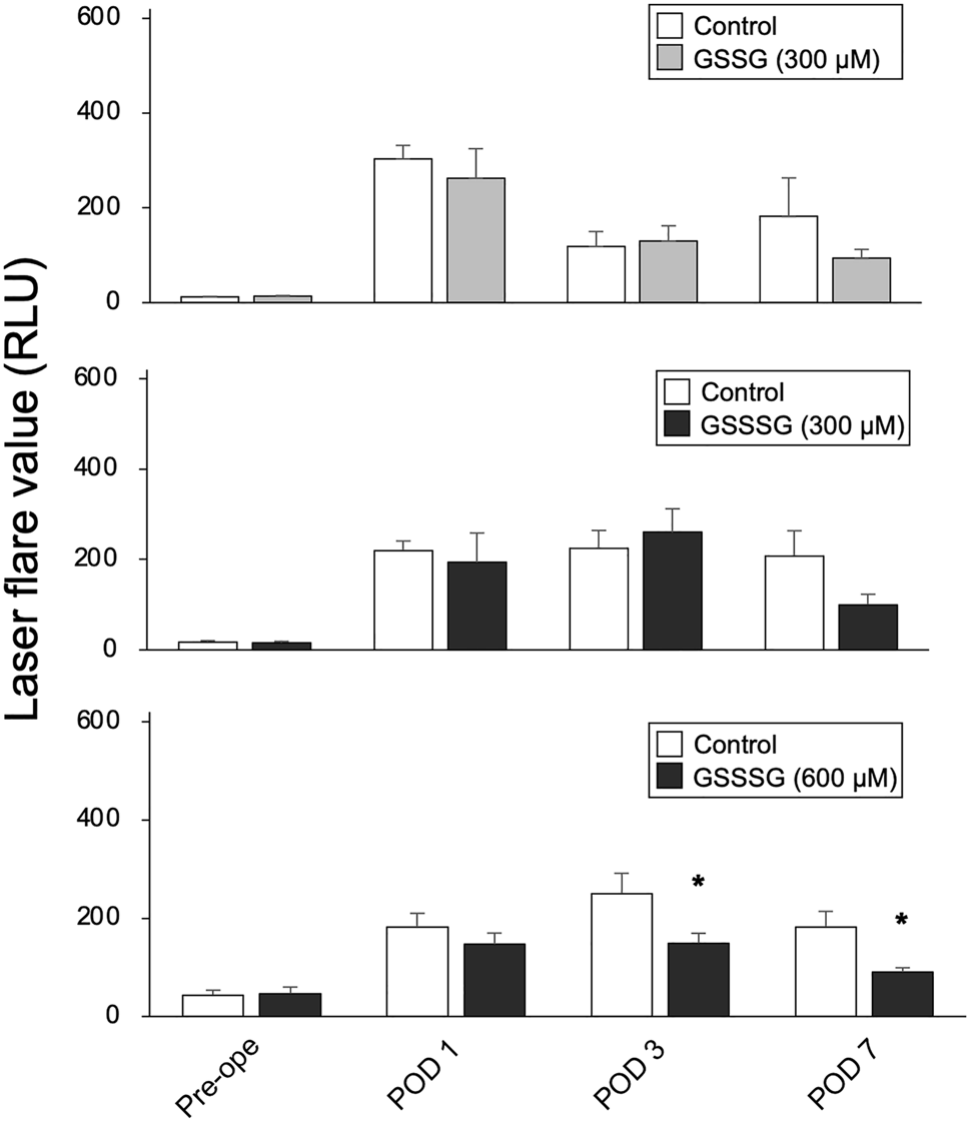
Effect of GSSSG on increased aqueous flare after cataract surgery. Time-course changes in aqueous flare in rabbit eyes before and after cataract surgery with irrigating solution containing GSSG (300 µM; top image), GSSSG (300 µM; middle image), or GSSSG (600 µM; bottom image). Error bars indicate standard error of the mean. *P < 0.05 (two-way repeated measures ANOVA; n = 4-9). RLU: relative light unit. Pre-ope: preoperative day. POD: postoperative day.

The rabbits underwent cataract surgery with an irrigating solution containing GSSSG (300 or 600 µM), GSSG (300 µM), or PB. Aqueous flare was examined with a laser flare meter on preoperative day and postoperative days 1, 3, and 7 (Fig. 2). On postoperative day 1, the flare value dramatically increased in the eyes of all the animals that had undergone cataract surgery (Fig. 2). The flare value significantly decreased in the eyes that had been irrigated with the GSSSG (600 µM)-containing solution on postoperative days 3 and 7, compared to the eyes irrigated with PB (Fig. 2). In contrast, there was no significant difference in flare value between the eyes irrigated with solutions containing GSSSG (300 µM) or GSSG (300 µM) and the corresponding controls at any time point (Fig. 2).

### GSSSG-mediated inhibition of ultrasonication-induced ROS

**Figure 3:**
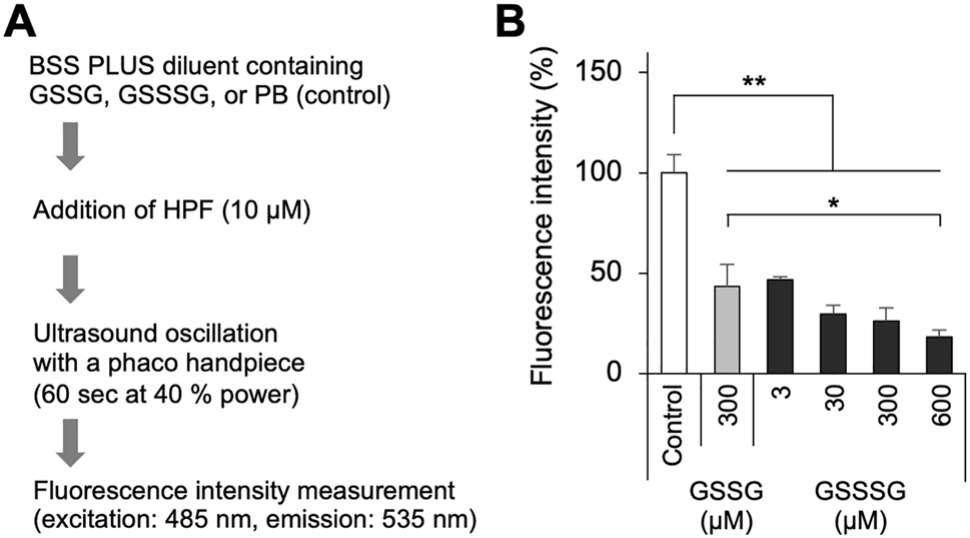
Effect of GSSSG on ultrasonication-induced ROS. (**A**) Experimental procedure for measurement of ultrasonication-induced ROS in glutathione-containing solutions treated with a phaco handpiece. (**B**) Effects of GSSSG and GSSG on ultrasonication-induced ROS. Fluorescence intensity is shown as a relative value to that of PB-containing control solution. Error bars indicate standard error of the mean. *P < 0.05 and **P < 0.01 (Dunnett’s test, Tukey–Kramer test; n = 4).

Finally, we examined the effects of GSSSG on ultrasonication-induced ROS using the ROS probe HPF (Fig. 3A). During phacoemulsification, longitudinal motions are made with the handpiece of the Alcon ACCURUS surgical system. Phacoemulsification generates free radical-mediated oxidative stress, which causes intraocular damage^4^. ROS generation was significantly prevented by the GSSG (300 µM)-containing solution compared to the control (Fig. 3B). GSSSG, as well as GSSG, exerted a similar inhibitory effect at various concentrations. GSSSG prevented the generation of ultrasonication-induced ROS in a dose-dependent manner (Fig. 3B). The level of generated ROS was significantly lower after application of the GSSSG (600 µM)-containing solution than the GSSG (300 µM)-containing solution (Fig. 3B).

## Discussion

To develop a more suitable IIS, we investigated the effect of GSSSG as a scavenger of ROS, and determined its protective effect in intraocular tissues. In an in vivo experiment, we performed irrigation with different IISs in rabbits, and found that leakage into the anterior chamber of FITC-labeled RSA was lower during perfusion with an IIS containing 300-μM GSSSG than with an IIS containing 300-μM GSSG. Furthermore, in vivo cataract surgery in rabbits revealed that aqueous flare was suppressed on days 3 and 7 after surgery with a 600-μM GSSSG IIS, but not at any period after surgery with a 300-μM GSSSG or 300-μM GSSG IIS. An in vitro experiment, without any live tissue, showed that ROS were suppressed more strongly by a 600-μM GSSSG IIS than a 300-μM GSSG IIS.

We found that there was a meaningful reduction of FITC-labeled RSA leakage into the anterior chamber during perfusion with a GSSSG IIS in our in vivo experiment; protein leakage is the result of surgically induced inflammation causing breakdown of the BAB^6,23^. The blood-ocular barrier comprises the BAB and the blood-retinal barrier (BRB). The BAB is formed by the non-pigmented epithelium of the ciliary body, the posterior iris epithelium, the endothelium of the iris vessels with tight junctions of the leaky type, and the endothelium of Schlemm’s canal^24^. The BAB is known to be relatively weak compared to the BRB^25,26^, and breakdown of the BAB can occur for various reasons, including acute inflammation due to intraocular surgery, ocular hypotony, and inflammatory mediators^24^. Intraocular microsurgery, including cataract surgery and vitreous surgery, has become much less invasive in recent years with the use of small-gauge surgical instruments and the elimination of sutures^27–36^. However, despite such advances, closed ocular surgery using IIS can still cause ocular inflammation and oxidative stress, leading to various adverse events^4,8,9,11–13,37^. Thus, the protective effect of the BAB during perfusion with a GSSSG IIS may allow us to reduce these postoperative ocular complications.

Aqueous flare intensity, an estimate of the aqueous protein level, was also suppressed postoperatively after cataract surgery with a GSSSG IIS, but not at any period after cataract surgery with GSSG IIS. Aqueous flare (light scattering due to proteins present in the aqueous humor) can be quantitatively measured with a laser flare meter. It is considered to be a non-invasive biomarker of inflammation and breakdown of the BAB, and is closely associated with ocular inflammatory cytokines and various ocular diseases^38^. In diabetic patients, the aqueous levels of vascular endothelial growth factor (VEGF) and interleukin-6 (IL-6) are significantly correlated with the severity of macular edema, as well as with aqueous flare intensity^39^. In Behçet’s disease, serum levels of interferon-γ and IL-27 are strongly correlated with aqueous flare values and cell counts^40^. The aqueous flare value is also significantly correlated with the severity of macular edema, as well as vitreous levels of VEGF and IL-6, in patients with retinal vein occlusion^41,42^. Furthermore, aqueous flare intensity is reported to be associated with various ocular conditions after intraocular surgery. The intensity has been reported to increase in patients with clinically significant cystoid macular edema after cataract surgery^10^. A higher level of anterior flare 1 week after cataract surgery has been shown to contribute to extensive postoperative contraction of the anterior capsule opening in diabetic patients, particularly those with diabetic retinopathy^43^. Elevated flare intensity has also been reported to be closely associated with the incidence of late postoperative proliferative vitreoretinopathy in patients with primary rhegmatogenous retinal detachment^44^. Thus, aqueous flare might be a promising biomarker to determine the need for prophylactic administration of anti-inflammatory drugs. However, suppression of aqueous flare after intraocular surgery with an IIS, preventing the need for additional pharmacological intervention, would be ideal, and may be clinically achievable with the use of a GSSSG IIS during intraocular surgery.

The strong suppression of ROS by the GSSSG IIS that we observed in our in vitro experiment is significant, because oxidative stress is mainly induced by ROS, which cause damage to intraocular cells by attacking nucleic acids, proteins, and lipids. ROS have been reported to be produced by free radicals during ultrasound oscillation in aqueous solutions; this process could lead to intraocular tissue damage^16,17^. Furthermore, ROS are associated with various diseases and are present in inflammatory infectious diseases, malignant tumors, metabolic disorders, and in ischemia/reperfusion injury^45–47^. ROS are closely associated with signal transduction via 8-nitroguanosine 3′,5′-cyclic monophosphate (8-nitro-cGMP), a secondary messenger of ROS signals^48,49^. Thus, ROS cause specific changes by impairing homeostasis of the redox signal, in addition to non-specific changes that are toxicity induced and level dependent. It is unclear what mechanism underlies the strong suppression of ROS by GSSSG, which does not occur via live cells or tissue. GSSSG with glutathione reductase (GSR) has been reported to generate GSSH, which leads to a marked ability to scavenge hydrogen peroxide (H_2_O_2_)^21^. In a previous report, a mixture of three different GS(S)nSGs, GSSSG (84%), glutathione tetrasulfide (GSSSSG) (15%), and glutathione pentasulfide (GSSSSSG) (1%) (i.e., excluding GSSG), increased retinal cell viability in the presence of H_2_O_2_ more strongly than GSSG^22^. Thus, glutathione polysulfides shows a ROS-quenching activity that is at least stronger than GSSG in the presence of GSR via conversion to its reduced form, i.e., GSSH, which is highly reactive. We believe that the current favorable results for using GSSSG to suppress ROS without GSR may reinforce the clinical application of GSSSG IISs. Recently, GSSSG has also been reported to attenuate inflammatory gene expression in retinal pigment epithelial cells via extracellular signal-regulated kinase signaling hyperactivation, independently of the NRF2/HMOX1 pathway^50^. Thus, GSSSG itself may prevent the occurrence of ocular diseases related to oxidative stress and inflammation.

When interpreting the current results for clinical use, a few limitations should be considered. First, this study had a small sample size and a short follow-up period. Second, we did not perform a density analysis of corneal endothelial cells (CECs), because rabbits have an innate ability to regenerate CECs, a critical difference with humans. After exposure to ultrasound from clinical phacoemulsification, the areas of damaged corneal endothelium in rabbits become re-covered with endothelial cells within 24 h of exposure, and corneal thickness returns to control levels in the same timeframe^51^. Thus, to confirm the efficacy of GSSSG as an IIS in humans, a CEC density evaluation should also be planned in a clinical trial. Furthermore, hydrogen has also been reported to prevent CEC damage during phacoemulsification in both animals and humans^3,52^, which might be an alternative option. However, since our results were obtained with a strict study design that used a new endogenous antioxidant system based on GSSSG (with a purity that can be beyond 98%), we believe that the current main findings, i.e., that IISs based on GSSSG had anti-inflammatory and anti-oxidative effects after intraocular surgery and may decrease the rate of surgical complications, can be considered reliable and will support future efforts to develop better IISs.

In conclusion, this is the first report to investigate the effect of GSSSG as a scavenger of ROS and its protective effect on intraocular tissue. An in vivo experiment in rabbits showed that leakage in the anterior chamber of FITC-labeled RSA was lower during perfusion with a 300-μM GSSSG IIS than with a 300-μM GSSG IIS. Furthermore, aqueous flare was suppressed 3 and 7 days after cataract surgery with a 600-μM GSSSG IIS, but not at any period after surgery with a 300-μM GSSSG or a 300-μM GSSG IIS. An in vitro experiment, without any live tissue, showed that ROS were suppressed more strongly by a 600-μM GSSSG IIS than a 300-μM GSSG IIS. Thus, this study found that novel IISs based on GSSSG had anti-inflammatory and anti-oxidative effects during and after intraocular surgery and may decrease the rate of complications. Further clinical investigation of IISs based on GSSSG is needed to confirm their protective effect on intraocular tissues.

## Data Availability

The datasets generated and analyzed during the current study are not publicly available, but are available from the corresponding author on reasonable request.

## References

[1] Flaxman, S. R. *et al.* Global causes of blindness and distance vision impairment 1990–2020: A systematic review and meta-analysis. *Lancet Glob. Health*. **5** (2017). 10.1016/S2214-109X(17)30393-5 29032195

[2] Cameron MD, Poyer JF, Aust SD (2001). Identification of free radicals produced during phacoemulsification. J. Cataract. Refract. Surg..

[3] Igarashi, T. *et al.* Hydrogen prevents corneal endothelial damage in phacoemulsification cataract surgery. *Sci. Rep*. **6** (2016). 10.1038/srep31190 PMC4976317 27498755

[4] Takahashi H (2002). Free radicals in phacoemulsification and aspiration procedures. Arch Ophthalmol..

[5] Glasser DB, Matsuda M, Ellis JG, Edelhauser HF (1985). Effects of intraocular irrigating solutions on the corneal endothelium after in vivo anterior chamber irrigation. Am. J. Ophthalmol..

[6] Araie M, Shirasawa E, Ohashi T (1990). Intraocular irrigating solutions and permeability of the blood-aqueous barrier. Arch Ophthalmol..

[7] Araie, M. & Kimura, M. Intraocular irrigating solutions and barrier function of retinal pigment epithelium. *Br. J. Ophthalmol*. **81** (1997). 10.1136/bjo.81.2.150 PMC1722121 9059251

[8] Shimazaki J, Amano S, Uno T, Maeda N, Yokoi N (2007). National survey on bullous keratopathy in Japan. Cornea.

[9] Suzuki, H., Oki, K., Takahashi, K., Shiwa, T. & Takahashi, H. Functional evaluation of corneal endothelium by combined measurement of corneal volume alteration and cell density after phacoemulsification. *J. Cataract. Refract. Surg*. **33** (2007). 10.1016/j.jcrs.2007.07.033 18053908

[10] Ersoy L, Caramoy A, Ristau T, Kirchhof B, Fauser S (2013). Aqueous flare is increased in patients with clinically significant cystoid macular oedema after cataract surgery. Br. J. Ophthalmol..

[11] Krarup T, Holm LM, la Cour M, Kjaerbo H (2014). Endothelial cell loss and refractive predictability in femtosecond laser-assisted cataract surgery compared with conventional cataract surgery. Acta Ophthalmol..

[12] Ji Y, Rong X, Lu Y (2018). Metabolic characterization of human aqueous humor in the cataract progression after pars plana vitrectomy. BMC Ophthalmol..

[13] Krarup T, Ejstrup R, Mortensen A, la Cour M, Holm LM (2019). Comparison of refractive predictability and endothelial cell loss in femtosecond laser-assisted cataract surgery and conventional phaco surgery: Prospective randomised trial with 6 months of follow-up. BMJ Open Ophthalmol..

[14] Galvis V, Tello A, Gutierrez AJ (2013). Human corneal endothelium regeneration: Effect of ROCK inhibitor. Invest. Ophthalmol. Vis. Sci..

[15] Ungricht, E. L. *et al.* Effect of longitudinal and torsional ultrasound on corneal endothelial cells: experimental study in rabbit eyes. *J. Cataract. Refract. Surg*. **48** (2022). 10.1097/j.jcrs.0000000000000737 PMC8712615 34224479

[16] Riesz P, Kondo T (1992). Free radical formation induced by ultrasound and its biological implications. Free Radic. Biol. Med..

[17] Murano N, Ishizaki M, Sato S, Fukuda Y, Takahashi H (2008). Corneal endothelial cell damage by free radicals associated with ultrasound oscillation. Arch Ophthalmol..

[18] Takahashi, H. Free radical development in phacoemulsification cataract surgery. *J. Nippon. Med. Sch*. **72** (2005). 10.1272/jnms.72.4 15834202

[19] Haimann MH, Abrams GW (1984). Prevention of lens opacification during diabetic vitrectomy. Ophthalmology.

[20] Manzanas L (2002). Oral flavonoids, chromocarb diethylamine salt and cyaninosides chloride, to eliminate lipoperoxidation postvitrectomy. Exp. Eye Res..

[21] Ida T (2014). Reactive cysteine persulfides and S-polythiolation regulate oxidative stress and redox signaling. Proc. Natl. Acad. Sci. USA.

[22] Kunikata H (2017). Metabolomic profiling of reactive persulfides and polysulfides in the aqueous and vitreous humors. Sci. Rep..

[23] Mishima S, Tanishima T, Masuda K (1985). Pathophysiology and pharmacology of intraocular surgery. Aust. N Z J. Ophthalmol..

[24] Occhiutto ML, Freitas FR, Maranhao RC, Costa VP (2012). Breakdown of the blood-ocular barrier as a strategy for the systemic use of nanosystems. Pharmaceutics.

[25] Cunha-Vaz JG (1976). The blood-retinal barriers. Doc. Ophthalmol..

[26] Green K, Pederson JE (1973). Effect of 1 -tetrahydrocannabinol on aqueous dynamics and ciliary body permeability in the rabbit. Exp. Eye Res..

[27] Wilczynski M, Supady E, Loba P, Synder A, Omulecki W (2011). Results of coaxial phacoemulsification through a 18-mm microincision in hard cataracts. Ophthalmic Surg. Lasers Imaging..

[28] Alio JL, Soria F, Abdou AA (2014). Femtosecond laser assisted cataract surgery followed by coaxial phacoemulsification or microincisional cataract surgery: Differences and advantages. Curr. Opin. Ophthalmol..

[29] He Q, Huang J, Xu Y, Han W (2019). Changes in total, anterior, and posterior corneal surface higher-order aberrations after 1.8 mm incision and 2.8 mm incision cataract surgery. J. Cataract Refract Surg..

[30] Gozawa, M. *et al.* Comparison of subconjunctival scarring after microincision vitrectomy surgery using 20-, 23-, 25- and 27-gauge systems in rabbits. *Acta Ophthalmol*. **95** (2017). 10.1111/aos.13459 28627080

[31] Kunikata H (2020). Successful surgical outcomes after 23-, 25- and 27-gauge vitrectomy without scleral encircling for giant retinal tear. Jpn. J. Ophthalmol..

[32] Kunikata H (2021). Retinal sensitivity and vessel density after macular hole surgery with the superior inverted internal limiting membrane flap technique. Retina.

[33] Kunikata H, Osada U, Abe T, Nakazawa T (2021). Efficacy of early microincision vitrectomy surgery in traumatic macular hole. Graefes Arch Clin. Exp. Ophthalmol..

[34] Osada U (2020). Association of retinal vessel density with retinal sensitivity in surgery for idiopathic epiretinal membrane. Graefes Arch Clin. Exp. Ophthalmol..

[35] Yui N, Kunikata H, Aizawa N, Nakazawa T (2019). Optical coherence tomography angiography assessment of the macular capillary plexus after surgery for macula-off rhegmatogenous retinal detachment. Graefes Arch Clin. Exp. Ophthalmol..

[36] Kunikata H, Abe T, Nakazawa T (2019). Historical, current and future approaches to surgery for rhegmatogenous retinal detachment. Tohoku J. Exp. Med..

[37] Takahashi H, Suzuki H, Shiwa T, Sakamoto A (2006). Alteration of free radical development by ophthalmic viscosurgical devices in phacoemulsification. J. Cataract Refract Surg..

[38] Tugal-Tutkun I, Herbort CP (2010). Laser flare photometry: a noninvasive, objective, and quantitative method to measure intraocular inflammation. Int. Ophthalmol..

[39] Funatsu H (2002). Increased levels of vascular endothelial growth factor and interleukin-6 in the aqueous humor of diabetics with macular edema. Am. J. Ophthalmol..

[40] Jiang S (2011). Serum levels of Th17-related cytokines in Behcet disease patients after cataract surgery. Mol. Vis..

[41] Noma H, Mimura T, Tatsugawa M, Shimada K (2013). Aqueous flare and inflammatory factors in macular edema with central retinal vein occlusion: a case series. BMC Ophthalmol..

[42] Noma H, Mimura T, Shimada K (2014). Role of inflammation in previously untreated macular edema with branch retinal vein occlusion. BMC Ophthalmol..

[43] Takamura Y (2013). Anterior capsule contraction and flare intensity in the early stages after cataract surgery in eyes with diabetic retinopathy. J. Cataract Refract Surg..

[44] Hoerster R (2013). Profibrotic cytokines in aqueous humour correlate with aqueous flare in patients with rhegmatogenous retinal detachment. Br. J. Ophthalmol..

[45] Safronova VG (2006). Generation of reactive oxygen species by umbilical blood cells and immune status of newborns at risk of infectious inflammatory diseases. Bull. Exp. Biol. Med..

[46] Snezhkina AV (2019). ROS generation and Antioxidant Defense Systems in Normal and Malignant Cells. Oxid. Med. Cell Longev..

[47] Szabo, M. R., Pipicz, M., Csont, T. & Csonka, C. Modulatory effect of myokines on reactive oxygen species in ischemia/reperfusion. *Int. J. Mol. Sci*. **21** (2020). 10.3390/ijms21249382 PMC7763329 33317180

[48] Sawa T (2007). Protein S-guanylation by the biological signal 8-nitroguanosine 3′,5′-cyclic monophosphate. Nat. Chem. Biol..

[49] Akaike T, Nishida M, Fujii S (2013). Regulation of redox signalling by an electrophilic cyclic nucleotide. J. Biochem..

[50] Tawarayama, H. *et al.* Glutathione trisulfide prevents lipopolysaccharide-induced inflammatory gene expression in retinal pigment epithelial cells. *Ocul. Immunol. Inflamm*, 1–12 (2020). 10.1080/09273948.2020.1833224 33215957

[51] Olson LE, Marshall J, Rice NS, Andrews R (1978). Effects of ultrasound on the corneal endothelium: II. The endothelial repair process. Br. J. Ophthalmol..

[52] Igarashi T (2019). Effects of hydrogen in prevention of corneal endothelial damage during phacoemulsification: A prospective randomized clinical trial. Am. J. Ophthalmol..

